# Hyperendemic Dengue and Possible Zika Circulation in the Westernmost Region of the Indonesian Archipelago

**DOI:** 10.3390/v14020219

**Published:** 2022-01-24

**Authors:** Harapan Harapan, Kritu Panta, Alice Michie, Timo Ernst, Suzi McCarthy, Muhsin Muhsin, Safarianti Safarianti, Tjut Mariam Zanaria, Mudatsir Mudatsir, R. Tedjo Sasmono, Allison Imrie

**Affiliations:** 1 Medical Research Unit, School of Medicine, Universitas Syiah Kuala, Banda Aceh 23111, Indonesia; harapan@unsyiah.ac.id (H.H.); mudatsir@unsyiah.ac.id (M.M.); 2 Tropical Disease Centre, School of Medicine, Universitas Syiah Kuala, Banda Aceh 23111, Indonesia; 3 Department of Microbiology, School of Medicine, Universitas Syiah Kuala, Banda Aceh 23111, Indonesia; 4 School of Biomedical Sciences, University of Western Australia, Nedlands, WA 6009, Australia; kritu.panta@uwa.edu.au (K.P.); alice.michie@uwa.edu.au (A.M.); timo.ernst@uwa.edu.au (T.E.); suzi.mccarthy@health.wa.gov.au (S.M.); 5 Pathwest Laboratory Medicine, Nedlands, WA 6009, Australia; 6 Department of Internal Medicine, School of Medicine, Universitas Syiah Kuala, Banda Aceh 23111, Indonesia; muhsin@unsyiah.ac.id; 7 Department of Parasitology, School of Medicine, Universitas Syiah Kuala, Banda Aceh 23111, Indonesia; safarianti@unsyiah.ac.id (S.S.); tjut.mariam@unsyiah.ac.id (T.M.Z.); 8 Eijkman Institute for Molecular Biology, Jakarta 10430, Indonesia; sasmono@eijkman.go.id

**Keywords:** dengue, Zika, seroprevalence, PRNT, Aceh, Indonesia

## Abstract

The transmission of dengue and other medically important mosquito-borne viruses in the westernmost region of Indonesia is not well described. We assessed dengue and Zika virus seroprevalence in Aceh province, the westernmost area of the Indonesian archipelago. Serum samples collected from 199 randomly sampled healthy residents of Aceh Jaya in 2017 were analyzed for neutralizing antibodies by plaque reduction neutralization test (PRNT). Almost all study participants (198/199; 99.5%) presented with multitypic profiles of neutralizing antibodies to two or more DENV serotypes, indicating transmission of multiple DENV in the region prior to 2017. All residents were exposed to one or more DENV serotypes by the age of 30 years. The highest geometric mean titers were measured for DENV-4, followed by DENV-1, DENV-2 and DENV-3. Among a subset of 116 sera, 27 neutralized ZIKV with a high stringency (20 with PRNT_90_ > 10 and 7 with PRNT_90_ > 40). This study showed that DENV is hyperendemic in the westernmost region of the Indonesian archipelago and suggested that ZIKV may have circulated prior to 2017.

## 1. Introduction

Dengue is endemic in Indonesia and was first reported in 1968 [1,2,3]. Dengue molecular epidemiology studies were conducted in major Indonesian cities and regions, including Bali [4], Jakarta [5], Jambi [6], Makassar [7], Semarang [8], Sukabumi [9], Surabaya [10], and Purwokerto [11]. These studies characterized circulating DENV serotypes, genotypes and lineages (reviewed in [3]). Understanding population seroprevalence data is also important, as an indicator of the magnitude of dengue infection and to inform dynamic transmission models. A recent assessment of sera collected during 2014 in Indonesia found that 98.6% of serum samples neutralized one or more DENV serotypes and 50.9% neutralized more than one serotype [12]. However few samples (23/776 sera; 3%) from the westernmost areas of Indonesia were included in the study, compared to other regions. We therefore undertook a seroprevalence assessment among randomly sampled residents of Ache Jaya to provide a comprehensive understanding of dengue transmission in this westernmost region of Indonesia.

Zika, caused by Zika virus (ZIKV), re-emerged as a public health threat in 2007, and multiple ZIKV outbreaks were subsequently reported [13,14,15,16,17]. Microcephaly associated with ZIKV infection was also reported in Asian countries [18,19,20]. The presence of ZIKV in Southeast Asia, prior to the 2007 re-emergence, was confirmed by detection of the virus in *Aedes aegypti* mosquitoes in Malaysia in 1966 [21]. Recent autochthonous transmission of ZIKV was confirmed with detection of cases in Thailand [22], the Philippines [23], Vietnam [24], Cambodia [25], Indonesia [26], Malaysia [27], as well as in travelers returning from a number of Southeast Asian countries [28]. However, only one large outbreak was reported in the region: in Singapore in 2016 [29]. It was postulated that the background herd immunity to ZIKV or cross-protection by antibodies against other flaviviruses may have prevented the development of large ZIKV outbreaks [30]. A recent study in Nicaragua revealed that recent DENV infection was significantly associated with a decreased risk of symptomatic ZIKV infection, although both prior and recent DENV infections did not affect the rate of total ZIKV infections [31].

Zika outbreaks were not reported in Indonesia, although there is evidence of ZIKV transmission [26,32,33,34,35,36]. Indonesia was ranked as the third country most at risk of ZIKV exposure due to its volume of airline travelers [37]. These studies indicate Zika is potentially a major health problem in Indonesia; however, relatively few studies assessed ZIKV seroprevalence in the country [34,38] and the number of samples included from Aceh province, the westernmost point of Indonesia, was very limited. Therefore, more data regarding ZIKV seroprevalence in Aceh are required to enhance our understanding of previous ZIKV transmission in this region. The aim of this study was to determine the prevalence of neutralizing antibodies (NAb) against DENV and ZIKV in residents of Aceh, Indonesia.

## 2. Materials and Methods

### 2.1. Serum Samples

A cross-sectional study was conducted in 2017 in two sub-districts within Aceh Jaya regency. Aceh Jaya is located 120 km south of the capital of Aceh province, Banda Aceh, and had a population of 89,618 residents in 2017 [39]. Dengue outbreaks were reported in Aceh regularly in each regency and Aceh Jaya has a similar pattern of dengue incidence to other regencies. Healthy residents, aged between 4 to 67 years old, who had resided for at least five years in the current regency were considered eligible for inclusion as participants of the study. Blood samples were collected and kept at −80 °C before use. Individuals with a chronic infection, autoimmune diseases or allergic diseases were excluded.

### 2.2. Viruses for PRNT

Two Indonesian DENV isolates were used as targets in the PRNT assay: DENV-1 (D1/IDN/Bali_005/2010, accession: KM216665) and DENV-2 (D2/IDN/Bali_070/2011). Both viruses were isolated from Western Australian (WA) travelers returning from Bali in 2010 [40] and in 2011, respectively. DENV-3 and DENV-4 WHO reference strains [41], DENV-3_H-87_ and DENV-4_H-241_, were selected for the assays. The prototype ZIKV Asian lineage, PRVABC59, a clinical isolate sampled in Puerto Rico in 2005, was used; this lineage was the first to be sampled in Indonesia [26].

### 2.3. DENV and ZIKV PRNT

The PRNT procedure, as described previously [42], was optimized for each virus with slight modifications. The serum samples were tested in a 24-well plate template using Vero cells (ATCC CCL-81). Cells were maintained in Dulbecco’s Modified Eagle Media (Thermo Fisher Scientific, Waltham, MA, USA), supplemented with 5% fetal bovine serum, 1% L-glutamine and 1% penicillin/streptomycin (Life Technologies, Carlsbad, CA, USA). Sera were heat-inactivated and two, serial, two-fold dilutions (1/10 and 1/40) were prepared using serum diluent (DMEM medium). Pre-titrated virus was mixed with respective plasma dilutions at an equal volume and incubated at 37 °C and 5% CO_2_. After incubation, 200 μL of plasma/virus mix was inoculated onto Vero monolayer and incubated for an hour. Following incubation, 1.2% methylcellulose was overlain on the assay and incubated for 4–14 days, until plaque formation was observed microscopically. Virus was fixed and plaques were visualized using methylene blue with 1% formalin. For each assay, a virus-only control and media-only controls were included for assay validation, and to enable the calculation of plaque reduction induced by the dilutions of sera, in comparison with the serum-free, virus control.

Plaques produced were manually counted, and the neutralization titer was expressed as the reciprocal value of the serum dilution. A nonlinear regression curve was fit and used to interpolate Nab titer at 50% and 90% reduction. A Tobit regression model with random effects was used [43]. As this was a largely explorative study, the serostatus of each sample was categorized using various criteria, a combination of two PRNT thresholds (PRNT_50_ and PRNT_90_) and two cut-offs of serum dilutions (10 and 40). The geometric mean titer (GMT), the mean PRNT titer, for each age group, gender and dengue serotype was calculated based on DENV PRNT results described previously [12].

### 2.4. PRNT Criteria

For dengue-specific PRNT profiles, sera were categorized as naïve, monotypic or multitypic based on the interpolated NAb titers at PRNT_50_ as follows: (a) naïve, if NAb titers were <10 for all four serotypes; (b) monotypic, if NAb titers were >10 for only one serotype *or* if titers were ≥10 for different serotypes, with a single serotype having a high titer (>80 and >5-fold higher than other serotype titers); and (c) multitypic, if the titers were ≥10 for different serotypes without a single predominant titer [12]. ZIKV infection was defined using criteria developed in the Indonesian context of endemic dengue [34], where ZIKV-specific PRNT_90_ was >4-fold higher than any anti-DENV NAb titer.

## 3. Results

### 3.1. Participants’ Characteristics

**Figure 1:**
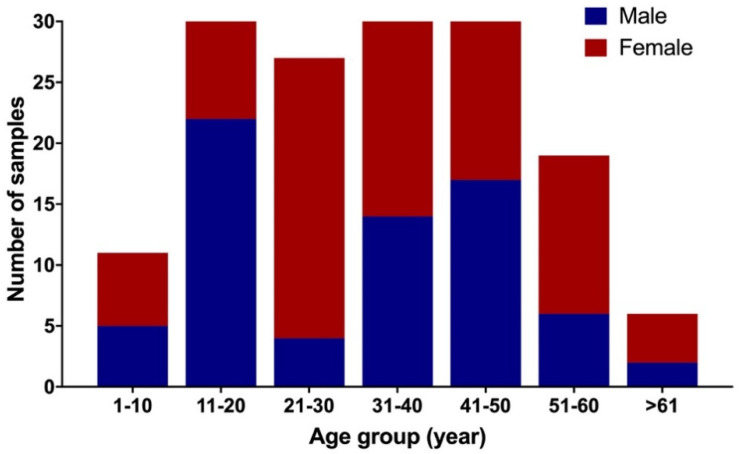
Number of participants based on age and gender (*n* = 199).

A total of 199 serum samples from the residents of Aceh were tested, of which 129 (64.8%) were female. The distribution of age groups and the gender of participants within each age group, is presented in Figure 1. The majority of the participants were aged between 11–50 years old. There were seven participants aged over 61 years old.

### 3.2. Prevalence of DENV Neutralizing Antibodies

PRNT seropositivity was defined with two thresholds, PRNT_50_ (50% reduction) and PRNT_90_ (90% reduction), and the above titers of 10 and 40. The proportion of anti-DENV NAb-positive individuals for each serotype is presented in Table 1. Using the standard cut-off point [12] of PRNT_50_ > 10, DENV NAb responses against at least one serotype were detected in all samples. Responses were highest against DENV-4 (100%), followed by DENV-1 (99.5%), while a slightly lower seropositivity was observed for DENV-3 (96.0%) and DENV-2 (99.0%). At the more stringent threshold of PRNT_90_ > 10, more than 75% of study volunteers had NAb responses against at least one serotype.

### 3.3. DENV Infection Status

**Figure 2:**
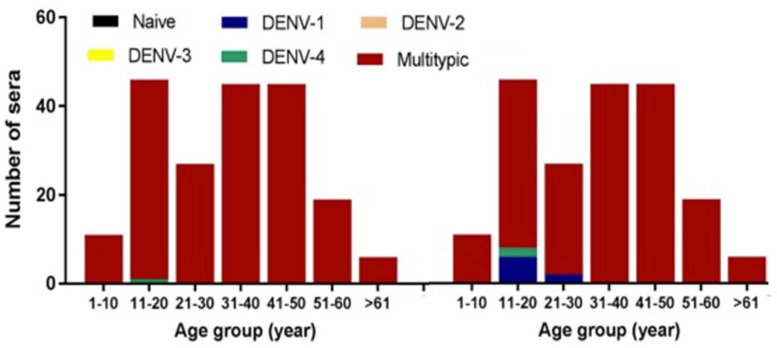
Status of DENV infection by age group (*n* = 199). Status of individuals with naïve, monotypic, or multitypic infections classified using two thresholds (PRNT_50_ and PRNT_90_ at >10). For each threshold, all monotypic infections were observed in individuals younger than 30 years old.

No samples demonstrated a naïve response of dengue, with NAb titers that were <10 for all four serotypes. Multitypic NAb responses were observed in 198/199 (99.5%) samples, and one sample had a monotypic DENV-4 response at threshold, PRNT_50_ > 10 (Figure 2). At a higher threshold, PRNT_90_ > 10, monotypic profiles were observed in eight samples for DENV-1 and two samples for DENV-4, all from individuals aged younger than 30 years old.

### 3.4. DENV NAb Geometric Mean Titer (GMT)

**Figure 3:**
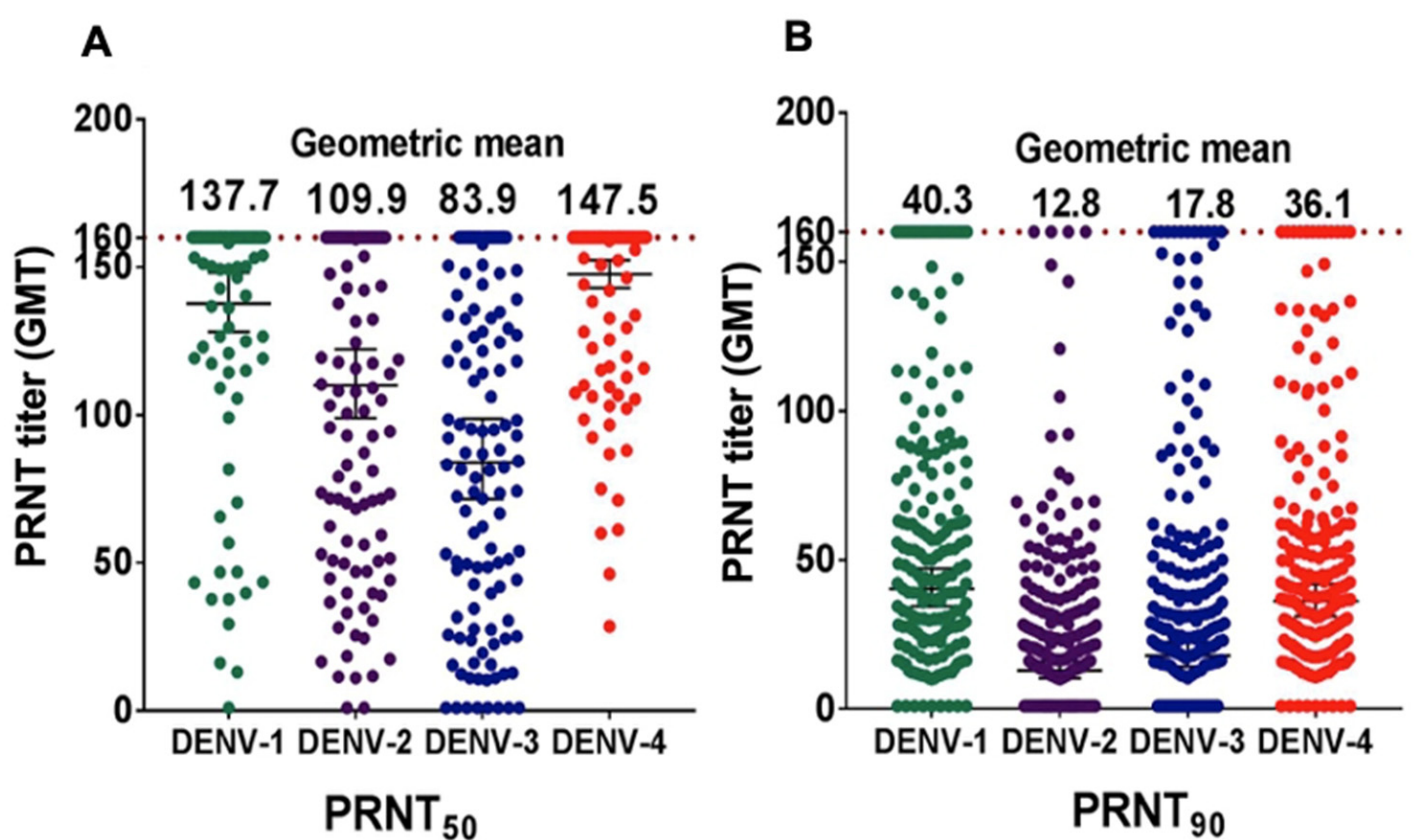
Geometric mean titers (GMT) of DENV NAb at two different thresholds: PRNT_50_ (**A**) and PRNT_90_ (**B**) (*n* = 199). Neutralizing antibody (NAb) GMTs were interpolated using non-linear regression.

Interpolated NAb titers were determined using a non-linear regression curve at two different thresholds, PRNT_50_ and PRNT_90_. NAb GMTs were calculated for each DENV serotype. At a PRNT_50_ threshold, DENV-4 had the highest GMT (147.5) followed by DENV-1 (137.7), DENV-2 (109.9) and DENV-3 (83.9) (Figure 3A). At a higher PRNT_90_ threshold, DENV-1 GMT was of the greatest magnitude followed by DENV-4, DENV-3 and DENV-2 (Figure 3B).

**Figure 4:**
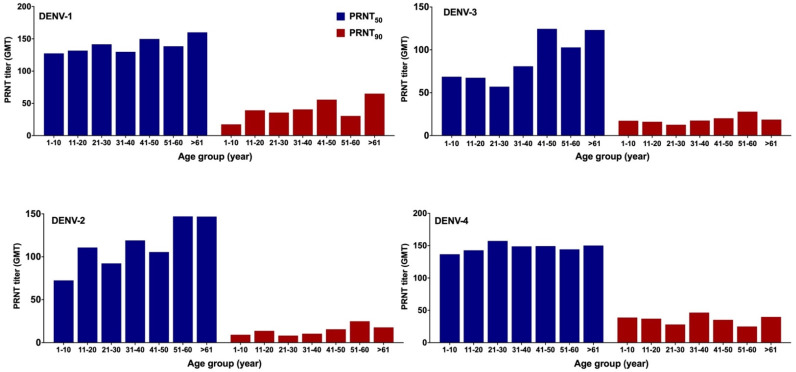
Stratification of geometric mean titer (GMT) of DENV neutralizing antibody (NAb) titers by age (*n* = 199). DENV NAb GMTs for each age group for all serotypes were calculated based on two thresholds PRNT_50_ (blue), and PRNT_90_ (red).

GMTs of NAb for each DENV serotype were stratified based on age and gender, which increased with age for all serotypes for both thresholds (Figure 4). This pattern, typically seen in dengue-endemic areas, was clearer for DENV-2 and DENV-3. At PRNT_50_, the GMTs for DENV-1 and DENV-4 were greater than 120 for all age groups (Figure 4). The GMTs were not influenced by gender for all four DENV serotypes. Although GMTs were slightly lower among females, in particular for DENV-1 and DENV-4, *t*-test analyses revealed that differences were not statistically significant (not shown).

### 3.5. Prevalence of ZIKV NAb

**Figure 5:**
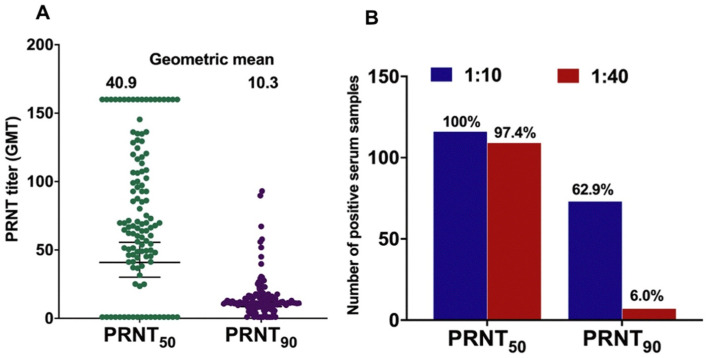
Geometric mean titer (GMT) of ZIKV neutralizing antibody (NAb) and the distribution of ZIKV NAb-positive sera at different thresholds (*n* = 116). (**A**) GMT of ZIKV NAb using two thresholds (PRNT_50_ and PRNT_90_). (**B**) ZIKV seropositive sera were classified using a combination of four cut-offs (two thresholds of plaque reductions percentage and two cut-offs for serum dilutions).

Sufficient volumes were available for 116/199 serum samples volume to assess ZIKV NAb responses. NAb GMTs at two thresholds (PRNT_50_, and PRNT_90_) are depicted in Figure 5A. ZIKV NAb GMTs were 40.9 and 10.3 for PRNT_50_ and PRNT_90_, respectively. The proportion of NAb-positive sera for each cut-off are presented in Figure 5B. At threshold PRNT_50_ > 10, NAb were detected in all specimens, while a total of 73 (62.9%) serum samples were seropositive at PRNT_90_ > 10, respectively. At the highest cut-off, PRNT_90_ > 40, 7 (6.0%) specimens were seropositive for ZIKV (Figure 5B).

### 3.6. GMT of ZIKV NAb

**Figure 6:**
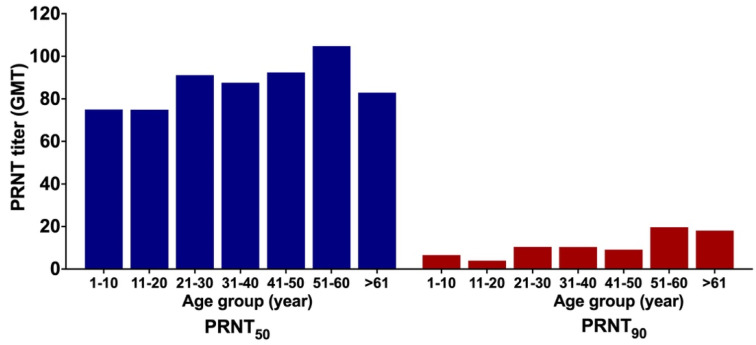
Geometric mean titers (GMTs) of ZIKV neutralizing antibody (NAb) at two thresholds (PRNT_50_ and PRNT_90_).

GMTs of ZIKV NAb were stratified based on age group and gender. The GMTs increased with age (Figure 6). At PRNT_50_, GMT for individuals aged 1–10 years old was 75.07 and peaked to 104.78 among those aged between 51 and 60 years. At PRNT_90_, GMT for individuals aged 1–10 years old was 6.63, increasing to 10.39 for the middle age group (31–40 years old), and to 18.12 for the oldest group (>61 years old) (Figure 6). There was no significant difference of GMT in ZIKV NAb titers between males and females for each threshold with *t*-test *p*-values of 0.343 and 0.223 for PRNT_50_ and PRNT_90_, respectively (Figure 6).

### 3.7. Confirmation of ZIKV Infection

**Figure 7:**
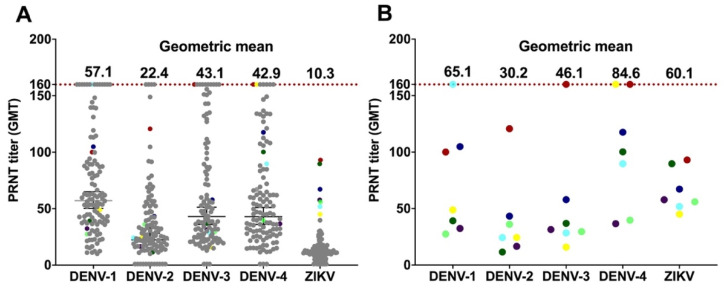
Neutralizing antibody (NAb) titers for ZIKV and DENV at PRNT_90_ (*n* = 116). (**A**) ZIKV NAb titers and corresponding DENV NAb titers were plotted to show dengue infection background. (**B**) For seven serum samples that had ZIKV NAb titers > 40, the DENV NAb titers were plotted individually and the GMTs were calculated. Using criteria developed in the Indonesian context [34] none of the samples can be classified as ZIKV-seropositive. One dot indicates an individual sample; grey dots indicate samples that have ZIKV NAb titers < 40 at PRNT_90_, while colored dots represent samples that had ZIKV NAb titers > 40 at PRNT_90_.

To confirm the validity of ZIKV infection among ZIKV-seropositive samples at the highest threshold (PRNT_90_), an additional confirmational step using stringent criteria was conducted. In Indonesia, due to the cross-reactivity between DENV and ZIKV, it was recommended that serum samples were classified: (a) ZIKV seropositive, if ZIKV PRNT_90_ titers > 4-fold higher than all DENV serotype PRNT_90_ titers; and (b) *Flavivirus* seropositive, if ZIKV NAb are present but at PRNT_90_ titers < 4-fold higher than any DENV PRNT_90_ titer [34]. The distribution of DENV NAb and ZIKV NAb titers at PRNT_90_ for all 116 serum samples and NAb GMTs for all four DENV serotypes and ZIKV are presented in Figure 7A. In this study, at the threshold PRNT_90_, 73 and 7 samples had ZIKV NAb titers > 10 and >40, respectively. Using these criteria, none of the samples were ZIKV-seropositive. However, 76 (65.5%) samples were classified as *Flavivirus* seropositive.

Detailed profiles of seven sera that had ZIKV NAb titers > 40 at PRNT_90_ were explored (Figure 7B and Table 2). When dengue infection background was assessed, all seven sera had multitypic anti-DENV NAb responses at PRNT_50_ > 10 (Table 2). However, at PRNT_90_ > 40, for both DENV and ZIKV, two of the samples, ID A-71 and A-119, were classified as naïve for DENV infection and seropositive for ZIKV, and thus suggested a ZIKV infection.

## 4. Discussion

### 4.1. Prevalence of DENV-Specific NAb

In this cross-sectional study, we assessed DENV seroprevalence among randomly sampled healthy inhabitants in Aceh Province, Indonesia using the gold standard plaque reduction neutralization test. Using a threshold PRNT_50_ > 10, all sera neutralized one or more DENV serotypes; 198 (99.4%) showed a multitypic profile and a single serum sample suggested prior monotypic infection with DENV-4. These data indicate dengue is hyperendemic in this westernmost region of Indonesia. The results from this present study are comparable with seroprevalence data among children (mean age of 9.6 years) from urban areas in Indonesia, in which 98.6% of 765 samples neutralized one or more DENV serotypes, with multitypic profiles in 50.9% of study participants [12]. In the present study, 99.4% of samples demonstrated multitypic DENV-neutralizing antibodies. This is unsurprising given the participants in the present study were older, with 75.2% aged 20 years or more, and were exposed to hyperendemic dengue transmission over their lifetimes.

In this present study, no monotypic DENV infections were observed in participants aged more than 30 years old. In addition, NAb GMTs increased with age indicating likely long-term dengue circulation in the area. This classic pattern was also observed in many studies in endemic and hyperendemic countries including India [44], Sri Lanka [45], Vietnam [46], and Mexico [47]. Our data confirm hyperendemic dengue circulation occurred for a significant period of time, likely many decades, in the westernmost region of Indonesia. A 1995 study in Yogyakarta, Central Java, found that 56.2% of 1837 children aged between 4–9 years old had PRNT_70_ NAb titers > 60. These data indicate that dengue was hyperendemic in different regions of Indonesia with intense transmission for decades, a trend that continued in recent years.

Data from this present study indicate that 100% of samples were positive for DENV-4 with fewer samples (191; 96%) seropositive for DENV-3. Across all four serotypes, DENV-4 demonstrated the highest NAb GMTs, and DENV-3 demonstrated the lowest, suggesting differential DENV serotype dominance within Aceh. However, serotype dominance could not be elucidated clearly as the proportion of monotypic infections was low. A previous study in 2014 found that, of 14 provinces assessed, DENV-4 predominance was observed in Aceh only, while in other provinces, DENV-1, DENV-2, and DENV-3 or a combination of these serotypes, were dominant, with DENV-2 dominance being more common [12]. Altogether, these findings indicate that the distribution of predominant serotypes varies by region in Indonesia. Given this diversity of dengue exposure history, it is unsurprising that the rates of symptomatic infection, and the pattern of dengue outbreaks, vary across Indonesian localities, although this is not entirely influenced by serotype distributions.

### 4.2. Prevalence of ZIKV-Specific NAb

Various thresholds were previously used to define ZIKV infection, for example PRNT_50_ ≥ 10 [48,49]. However, the high degree of structural and nucleotide sequence homology between ZIKV and other flaviviruses [50] means that distinguishing ZIKV infection in samples from dengue-hyperendemic regions, such as Aceh, is challenging particularly if samples are collected from adults. The WHO recommend the use of the PRNT_90_ threshold as the gold standard confirmatory assay for ZIKV serology [51], and previous studies used endpoints of PRNT_90_ ≥ 10 [52,53,54] or ≥20 [55,56] to define the ZIKV seropositivity. In our study, at threshold PRNT_90_ > 10 and >40, there were 75 (64.6%) and 7 (6.0%) samples classified as ZIKV-seropositive. However, as serum samples used in this study had continuous exposure to DENV, interpretation was more complex.

Using a previously defined criterion for assessing ZIKV seropositivity in Indonesia, ZIKV-specific PRNT_90_ were > 4-fold higher than any anti-DENV NAb titer [34]; none of the serum samples were positive for ZIKV infection. Nevertheless, more than 75% of the specimens were *Flavivirus*-seropositive. One reason for this high proportion of nonspecific *Flavivirus* seropositivity is the large proportion of older participants in this study, with 75.2% of participants being aged 20 years old or older and 100% of the participants exposed to at least one DENV serotype. Nevertheless, by using stringent criteria of PRNT_90_ > 40, the possibility of ZIKV infection could not be excluded for samples A-71 and A-119 (Table 2). If the same PRNT_90_ > 40 threshold was used to characterize ZIKV and DENV infections, these two samples would be classified as naïve for all four DENV serotypes and seropositive for ZIKV, suggesting a true ZIKV infection. Other assays to distinguish between ZIKV and DENV antibodies were not available for the present study; we aim to develop more specific protocols in the near future.

The possibility of DENV and ZIKV co-infection cannot be ruled out. In one study, when ZIKV NAb titers were measured 12–19 months after illness onset among PCR-confirmed Zika cases, only 70.1% of 62 samples could be characterized as ZIKV infections based on the described criteria [57]. Furthermore, five (8%) samples had DENV NAb titers > 4-fold higher than ZIKV NAb titers [57]. Due to this fact, diagnosing ZIKV based on the criteria of a ZIKV PRNT_90_ titer being > 4-fold higher than that of any DENV NAb titer potentially underreports Zika cases in dengue endemic areas.

The cross-reactivity between ZIKV and DENV is not only a diagnostic dilemma but may also have clinical consequences. DENV-induced antibodies were shown to enhance ZIKV infection in FcγR-bearing monocytic cell lines [58]. In addition, other studies reported that anti-DENV antibodies may enhance ZIKV infection through the process of antibody-dependent enhancement (ADE) of infection [59,60,61,62]. Although growing evidence also suggests that prior DENV has a protective role in ZIKV infection [31,63,64,65], studies in rhesus macaques [66,67] and humans [68] do not support ZIKV enhancement. Other studies suggested that protection or enhancement in ZIKV infection may depend upon the level of anti-DENV antibodies present [59]. 

### 4.3. Study Limitations

In this study, most serum samples were collected from participants aged five years old or older. This means that most samples had multitypic infection profiles; therefore, it was difficult to determine the current predominant serotypes in circulation. Therefore, the analysis of samples from a younger population is required for better discrimination of predominant DENV serotypes. In addition, limited molecular data of previous ZIKV infections means we are unable to describe ZIKV transmission in this region.

## 5. Conclusions

All four DENV serotypes circulated in Aceh prior to 2017. DENV-4 seropositivity was identified at the highest frequency in the present study and the highest mean GMT titers were measured for DENV-4 followed by DENV-1, DENV-2 and DENV-3; however, the small sample size limits any generalization to DENV-4 seroprevalence in Aceh. All respondents were exposed to one or more DENV serotypes by the age of 30 years. This indicates DENV is hyperendemic in Aceh. Among 116 sera that had a sufficient volume for analysis, 20 were reactive with ZIKV at a threshold of PRNT_90_ > 10 and 7 at PRNT_90_ > 40. However, when ZIKV seropositivity was defined as ZIKV-specific PRNT_90_ > 4-fold higher than any anti-DENV NAb titer, none of the samples could be classified as ZIKV-seropositive; however, 76 (65.5%) of samples were classified as *Flavivirus*-seropositive.

## Figures and Tables

**Table 1:** Proportion of DENV NAb-positive sera at different thresholds (*n* = 199).

Threshold and Titer Cut-Off	DENV-1 *n* (%)	DENV-2 *n* (%)	DENV-3 *n* (%)	DENV-4 *n* (%)
PRNT_50_				
10	198 (99.5)	197 (99.0)	191 (96.0)	199 (100.0)
40	192 (96.5)	181 (91.0)	168 (84.4)	198 (99.5)
PRNT_90_				
10	190 (95.5)	151 (75.9)	156 (78.4)	190 (95.5)
40	111 (55.8)	45 (22.6)	68 (34.2)	97 (48.7)

**Table 2:** Comparison of DENV and ZIKV NAb-positive sera for (*n* = 7).

ID	Age (Year)	PRNT_90_ Titer for DENV				Status of Dengue Infection		ZIKV PRNT_90_ Titer	Infection Status *
		DENV-1	DENV-2	DENV-3	DENV-4	PRNT_90_ > 10	PRNT_90_ > 40		
A-157	53	100.05	120.75	>160	>160	Multitypic	Multitypic	93.119	*Flavivirus*
A-52	63	39.19	11.55	36.86	100.23	Multitypic	DENV-4	89.746	*Flavivirus*
A-97	60	104.86	43.27	57.94	117.66	Multitypic	Multitypic	67.247	*Flavivirus*
A-71	53	32.43	16.61	31.40	36.67	Multitypic	Naïve	57.835	*Flavivirus*
A-119	22	27.48	36.11	29.55	39.67	Multitypic	Naïve	55.986	*Flavivirus*
A-94	50	>160	24.31	28.42	89.72	Multitypic	Multitypic	51.872	*Flavivirus*
A-13	55	48.83	24.40	15.89	160.00	Multitypic	DENV-4	45.076	*Flavivirus*

* ZIKV seropositive: ZIKV PRNT_90_ titers > 4-fold higher than all DENV serotype. *Flavivirus* seropositive: ZIKV NAb are present but at titers < 4-fold higher than any DENV NAb titer [34].

## Data Availability

The NAb titers of samples are available from the corresponding author upon reasonable request.
